# DNA Double Strand Breaks but Not Interstrand Crosslinks Prevent Progress through Meiosis in Fully Grown Mouse Oocytes

**DOI:** 10.1371/journal.pone.0043875

**Published:** 2012-08-22

**Authors:** Wai Shan Yuen, Julie A. Merriman, Moira K. O'Bryan, Keith T Jones

**Affiliations:** 1 School of Biomedical Sciences and Pharmacy, University of Newcastle, Callaghan, New South Wales, Australia; 2 Department of Anatomy and Developmental Biology, Monash University, Clayton, Victoria, Australia; University of Massachusetts Medical School, United States of America

## Abstract

There is some interest in how mammalian oocytes respond to different types of DNA damage because of the increasing expectation of fertility preservation in women undergoing chemotherapy. Double strand breaks (DSBs) induced by ionizing radiation and agents such as neocarzinostatin (NCS), and interstrand crosslinks (ICLs) induced by alkylating agents such as mitomycin C (MMC), are toxic DNA lesions that need to be repaired for cell survival. Here we examined the effects of NCS and MMC treatment on oocytes collected from antral follicles in mice, because potentially such oocytes are readily collected from ovaries and do not need to be in vitro grown to achieve meiotic competency. We found that oocytes were sensitive to NCS, such that this ionizing radiation mimetic blocked meiosis I and caused fragmented DNA. In contrast, MMC had no impact on the completion of either meiosis I or II, even at extremely high doses. However, oocytes treated with MMC did show γ-H2AX foci and following their in vitro maturation and parthenogenetic activation the development of the subsequent embryos was severely compromised. Addition of MMC to 1-cell embryos caused a similarly poor level of development, demonstrating oocytes have eventual sensitivity to this ICL-inducing agent but this does not occur during their meiotic division. In oocytes, the association of Fanconi Anemia protein, FANCD2, with sites of ICL lesions was not apparent until entry into the embryonic cell cycle. In conclusion, meiotic maturation of oocytes is sensitive to DSBs but not ICLs. The ability of oocytes to tolerate severe ICL damage and yet complete meiosis, means that this type of DNA lesion goes unrepaired in oocytes but impacts on subsequent embryo quality.

## Introduction

Mammalian oocytes spend most of their lives at the dictyate stage of meiosis I, in so-called Germinal Vesicle (GV) arrest. Meiotic resumption is only initiated under a hormonal cue in the hours preceding ovulation. This unique type of cell arrest however presents problems in maintaining oocyte health over the months, and dependent on species, possibly decades of cell cycle arrest. In part, this problem is solved by a 2-way communication of growth factors with surrounding granulosa cells within the follicle [1], [2], [3]. Despite this somatic support, however, female aging still results in a drop in oocyte quality. Most notably, this is observed by a rise in mis-segregation of homologous chromosomes in meiosis I [4], [5], possibly caused by a loss in the cohesive proteins holding them together [6], [7], [8]. In oocytes, cohesin complexes get loaded onto replicating chromosomes during fetal life but cannot be repaired in the adult [9], [10]. As such with age, homologous chromosomes loose attachment with each other.

Given that oocytes spend the majority of their long life in GV arrest, it is important to resolve what their capacity is to detect and respond to damage done to the DNA itself, as opposed to the proteins holding chromosomes together. Especially considering the increasing expectation for fertility preservation in females of reproductive age and younger, undergoing cancer treatment [11], [12]. GV oocytes contained with primordial follicles, which represent the longest stage of meiotic arrest, die in response to double strand breaks (DSBs) induced by ionising radiation (IR) [13], [14], probably through a c-Abl-p63 mediated apoptotic pathway, which appears to be most active in non-growing follicles [15], [16], [17]. GV bovine oocytes contained within growing or fully mature follicles have also been suggested to undergo cell death in response to IR [18], and similarly meiosis stalls in pig oocytes from mature follicle that are exposed to UV-C light [19].

Interstrand crosslinks (ICLs) are extremely toxic lesions, distinct from DSBs, formed between 2 strands of DNA. Without repair, they prevent DNA replication [20]. Around 10 ICLs are believed to be formed per cell per day in somatic cells [21]. Agents such as mitomycin C (MMC) that chemically induce ICLs, are an effective chemotherapeutic agent to help prevent proliferation of cancer cells and are a useful tool to study the consequences of ICLs on cell function. The Fanconi Anemia (FA) pathway is thought to play an important part in ICL detection and repair [22], [23], [24], and its activation recruits FANCD2 and FANCI together to form the so-called ID complex, at discrete nuclear foci of DNA damage. Some members of the FA pathway, including the ID complex, load onto site of ICLs independent of replication [25], [26], suggesting that FANCD2, as part of the ID complex, is involved at an early step in the sensing of an ICL.

Given the importance of the FA pathway in ICL DNA repair, and possibly more generally in metabolic-induced DNA damage through production of acetaldehydes [27] and oxidative stress [28], [29], here we decided to examine the timing of ICL DNA repair and FA activation in mouse GV oocytes and preimplantation embryos. Specifically, we wanted to examine if oocytes have the capacity to sense DNA damage induced by MMC, as assessed by the targeting of FANCD2 to site of ICLs. We thought it important to examine from this time onwards because clinically these are the stages used for assisted fertility treatments and it would be useful to understand how these oocytes respond to increased ICL DNA damage. The sensitivity of mouse oocytes to DNA damage brought about by MMC-induced ICLs was compared to that of DSBs caused by the widely used ionizing radiation mimetic neocarzinostatin (NCS) [30], [31], [32], [33].

## Materials and Methods

### HeLa Cell Culture

HeLa cells (ATCC, Cat No. CCL-2) were cultured in DMEM Low Glucose (Invitrogen) supplemented with 10% FCS (Invitrogen) and 1% penicillin-streptomycin (Invitrogen) at 37°C in 5% CO_2_. A double thymidine block was performed as described previously [34].

### Ethics Statement

Mice were used in strict accordance with the Australian Code of Practice for the Care and Use of Animals for Scientific Purposes. This protocol was approved by the University of Newcastle Animal Care and Ethics Committee (Ethics approval number A-2008-1079).

### Oocyte Collection and Culture

GV oocytes and mature eggs were collected from hormonally primed F1 hybrid mice (C57BL6 females × CBA males) as described previously [35], [36]. Milrinone (1 μM) was added to maintain arrest [37]. GV oocytes were allowed to mature in vitro for 14 hours in M2 media.

### Embryo Activation and Culture

Mature eggs were parthenogenetically activated in Ca^2+^-free KSOM medium supplemented with 10 mM SrCl_2_ for 5 hours [38]. 1 µg/ml of cytochalasin D was added to diploidise embryos. 1-cell embryos were treated at 5 hours post-activation with MMC for 4 hours. Embryo culture was in KSOMaa [39] at 37°C in 5% CO_2_. Pronuclei, 2-cell embryos and blastocysts were scored at 9, 24 and 120 hours post-activation respectively.

### Cell, Oocyte and Embryo Treatments

In HeLa cells, 250 ng/ml MMC was added immediately after thymidine release for 4 hours and cells were fixed either at 4 or 7 hours post release. In oocytes, MMC at specified concentrations were added immediately after milrinone washout for 4 hours and cells were fixed either after maturation or at 9 hours post activation. NCS at specified concentrations was added immediately after milrinone washout for 1 hour, and oocytes were fixed following in vitro maturation. In embryos, MMC was added at 5 hours post-activation for 4 hours and embryos were fixed at 9 hours post-activation.

### Immunofluorescence

HeLa cells, cultured on coverslips, were fixed in 4% paraformaldehyde in PHEM/PVP then permeabilised with 2% Triton X-100, 30 minutes each at room temperature. Oocytes and embryos were fixed and permeabilised as described previously [40]. Immunofluorescence was performed as described previously [41] using a monoclonal anti-tubulin antibody (1∶200, Molecular Probes) and/or polyclonal anti-FANCD2 antibody (1∶500, Abcam) or anti-γH2AX antibody (1∶100, Abcam) supplemented with 1% BSA and 0.2% Tween 20 for 1–2 hours at room temperature. Secondary antibodies, conjugated with Alexa488 or Alexa633 (1∶1000, Molecular Probes), were used for detection by incubating cells for 1 hour at room temperature. Oocytes or HeLa cells were briefly stained with Hoechst (20 μg/ml) or propidium iodide (10 µg/ml) to label chromatin before mounting on glass slides with Citifluor (Citifluor Ltd, UK).

### Imaging and Data analysis

Confocal microscopy was performed using an Olympus FV1000 equipped with a 60x/1.2 NA UPLSAPO oil immersion objective lens. Z-stacks were captured with an interval of 1–2 μm. Analysis was performed with FV10-ASW 2.0 Viewer software (Olympus). For foci counting, images were converted to 8-bit gray images and foci were counted using ImageJ software (NIH, Bethesda, USA) following setting their threshold to 1.7 times the average background. This value of 1.7 was used because it most closely matched foci counted by users in initial somatic cell experiments.

### Statistical analysis

All statistical analysis was performed using Graphpad Prism 5.0 software. Dichotomous data, such as polar body extrusion rates, were pooled from repeated observations. Comparison between groups was made using Fisher's exact test. FANCD2 foci data were pooled and means between 2 groups compared by Student's t-test or by ANOVA for multiple groups. The number of repeats is stated in each figure legend.

## Results

### Neocarzinostatin blocks oocyte maturation and fragments DNA

**Figure 1:**
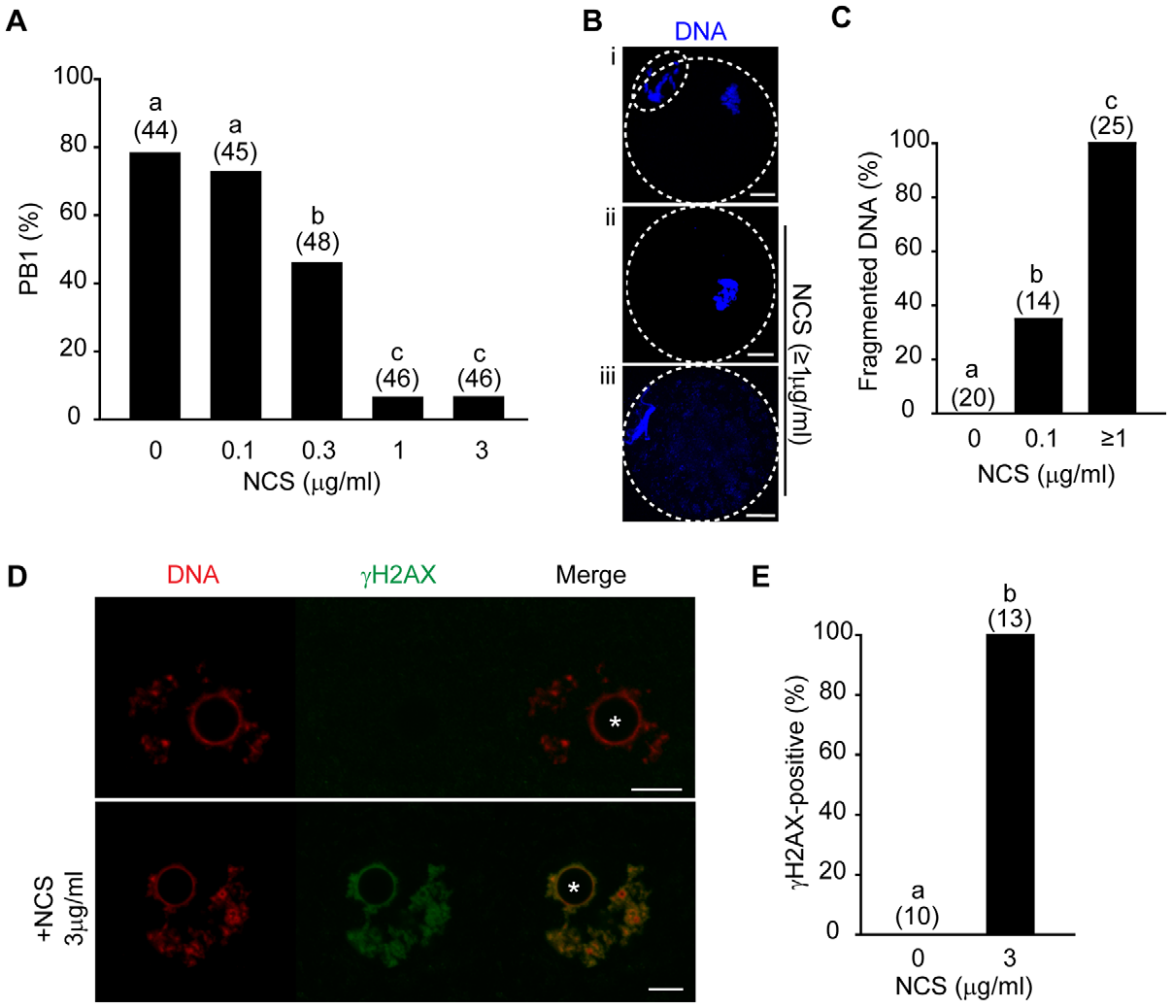
Neocarzinostatin, an ionizing radiation mimetic, fragments DNA and blocks meiosis in oocytes. (A) First polar body (PB1) extrusion rates following maturation of oocytes treated with NCS. (B) Chromatin in oocytes from (A); (i) depicting a typical metaphase II egg that was not treated with NCS, (ii, iii) meiotic arrest caused by NCS with varying levels of fragmentation. White dotted line represents egg and polar body outlines. (C) Percentage of oocytes treated with or without NCS that display fragmented DNA. (D) Nuclear staining of γH2AX in GV oocytes with or without NCS treatment; asterisks is at center of nucleolus. (E) Percentage of γH2AX positive oocytes treated with or without NCS. (A, C, E) Pooled data from 2 replicates. In parentheses, total number of oocytes examined. (A) Different letters denote significant difference, p<0.05 (Fisher's exact test). (B, D) Scale bar, 10 µm.

Mouse GV stage oocytes collected from antral follicles were treated with the ionizing radiation mimetic NCS for a period of 1 hour before in vitro maturation. They were allowed to mature through to metaphase II (metII) and first polar body extrusion (PB1) rates assessed. Maturation rates were normally ∼80%, however significant inhibition of meiosis I was observed at NCS concentrations of 0.3 µg/ml and greater (Fig 1A). Furthermore, the rate of PB1 extrusion was exceedingly low at ∼7% following a 1 hour incubation of GV oocytes with 1 µg/ml NCS. Examination of the DNA from meiotically arrested oocytes treated with NCS showed condensed and varying levels of fragmented chromatin, with fragmentation high in a number of oocytes (Fig 1B). The effects of NCS appeared dose dependent, with 35% of oocytes having such fragmentation at 0.1 μg/ml, rising to 100% at higher doses (1–3 μg/ml); none of the untreated oocytes displayed such abnormalities (Fig 1C).

Ataxia Telangiectasia Mutated (ATM) and ATM-and Rad3-related (ATR), are major regulators of DNA damage, and phosphorylate the histone H2AX, generating the epitope recognised by anti- γH2AX antibodies [42], [43], [44]. We therefore immunoprobed GV oocytes, and found very significant amounts of γH2AX foci associated with chromatin in all oocytes following incubation with NCS; in contrast, all untreated oocytes did not stain positive for γH2AX (Fig 1D, E).

### Mitomycin C-induced ICLs do not block completion of meiosis I or II in oocytes

**Figure 2:**
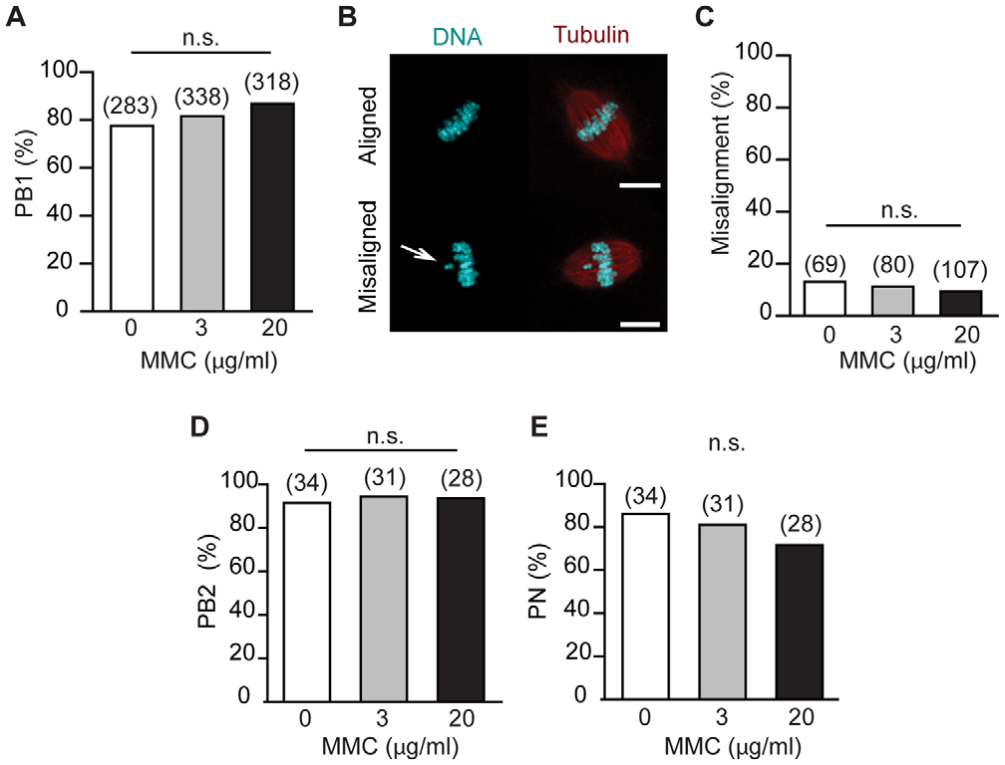
Interstrand crosslinking has no effect on completion of meiosis in oocytes. (A) First polar body (PB1) extrusion rates in oocyes treated with MMC after milrinone washout. (B, C) Meiotic spindles from mature metaphase II stage eggs obtained in (A) were imaged (B) and the sister chromatids on the spindle classified as aligned or mis-aligned (C). Arrow, indicates a non-aligned chromosome. Scale bar, 10 μm. (D, E) Rates of second polar body extrusion (D) and pronucleus formation (E) in parthenogenetically activated eggs following addition of MMC to GV stage oocytes that were then in vitro matured. (A, C, D, E) Pooled data from between 4 to 5 replicates; in parentheses, number of eggs examined; n.s., p>0.05 (Fisher's exact test).

In order to begin to understand the effect that ICLs have on meiosis, GV oocytes were in vitro cultured with MMC added for the first 4 hours. PB1 extrusion rates were determined 8 hours later. MMC had no effect on the ability of oocytes to undergo meiotic maturation, with polar body rates high at about 80% for all groups (Fig 2A). The highest concentration of MMC tested was 20 µg/ml, which is ∼1000-fold higher than reported to be effective on mitotic cells [45] and this had no statistical significance on first polar body extrusion rate compared to controls (86% vs 77%, p = 0.13, n.s. Fig 2A).

The effect that MMC has on nuclear maturation of oocytes may be subtler than simply blocking the process of PB1 extrusion. Therefore, the chromatin of metII eggs matured from GV oocytes that had been treated with MMC were examined for meiotic spindle integrity. However, we observed no difference in the meiotic spindles from MMC-treated eggs, with small rates of chromosomal misalignment for all groups (Fig 2B, C). Finally, we examined their ability to complete meiosis and become 1-cell embryos by parthenogenetic activation with Sr^2+^-containing medium. The rates of second polar body (PB2) extrusion and pronucleus (PN) formation rates were found to be unaffected by previous MMC addition (Fig 2D, E). From all these observations we can conclude that the induction of ICLs during the first 4 hours of maturation has no inhibitory effect on the ability of oocyte to complete its meiotic divisions and become a 1-cell embryo.

### Mitomycin C-induced ICLs inhibit embryo development

**Figure 3:**
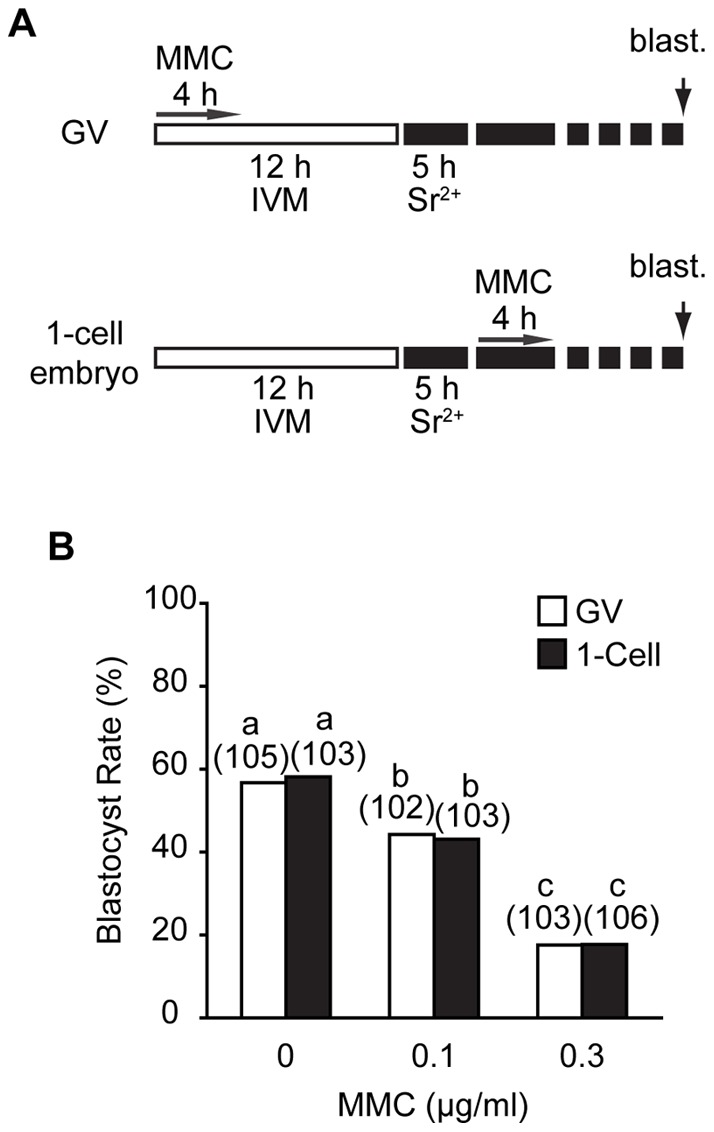
Comparable impaired embryo development following ICL induction at either GV or 1-embryo stage. (A) Schematic showing the experimental design. MMC was either added after milrinone washout or following parthenogenetic activation with Sr^2+^. Blastocyst rates were assessed at 120 hours. (B) Blastocyst rates from those oocytes treated as in (A) with the MMC concentration stated. In parentheses, number of eggs examined from 3 pooled replicates; different letters denote significantly difference (p<0.05; Fisher's exact test).

Next, we wanted to establish if the induction of ICLs at the GV stage has an impact on subsequent embryo development. To examine this, GV oocytes were treated with the same two doses used above, 3 or 20 μg/ml MMC for the first 4 hours of maturation, in vitro matured, parthenogenetically activated, and preimplantation embryo development to the blastocyst stage assessed (Fig 3A). For GV oocytes cultured without MMC, we observed nearly 60% rates of development to the blastocyst stage. However, less than 7% of embryos developed past the morula stage if they had been pre-treated with either of the two MMC concentrations (not shown). In order to observe any development to the blastocyst stage, the concentration of MMC needed to be lowered. GV oocytes treated with 0.1 µg/ml MMC were found to have a significantly reduced blastocyst formation rate compared to controls (57% vs 44%, p = 0.03, χ^2^ Fisher's exact test) and 0.3 μg/ml MMC reduced development to below 20% (Fig 3B).

Finally we thought it would be interesting to determine if exposure of GV oocytes to MMC was in any way more or less detrimental than if 1-cell embryos were incubated with this chemotherapeutic agent. In both groups, GV oocytes were in vitro matured, parthenogenetically activated and cultured to the blastocyst stage. However, for those 1-cell embryos treated with MMC, its incubation followed pronucleus formation at 5 hours after parthenogenetic activation (Fig 3A). With the two MMC doses examined, we observed no significant difference in the rate of blastocyst formation (Fig 3B) between those exposed at the GV stage and those exposed as 1-cell embryos.

In summary, the above data suggest that induction of ICLs in GV stage oocytes has no impact on the completion of meiosis but does impact on subsequent development to the blastocyst stage. It was interesting that even at doses 200-fold higher than that able to impair embryogenesis the process of meiosis was unaffected. The observation that oocytes treated at the GV stage with MMC had severely impacted embryo development following their in vitro maturation (Fig 3) also highlights the fact that any lack of effect of MMC during maturation was not simply a consequence of GV oocytes being impermeant to this drug. In fact, GV oocytes and 1-cell embryos are likely to be equally permeant to this ICL-inducing agent, given rates of embryo development were affected to the same degree when MMC was added to them. Thus, as judged by the inhibition of blastocyst formation, the developmental time point for MMC addition appears unimportant as similar inhibition rates were observed whether MMC was added at the GV stage or in 1-cell embryos.

### ICL induced FANCD2 nuclear foci in somatic cells but not in oocytes

**Figure 4:**
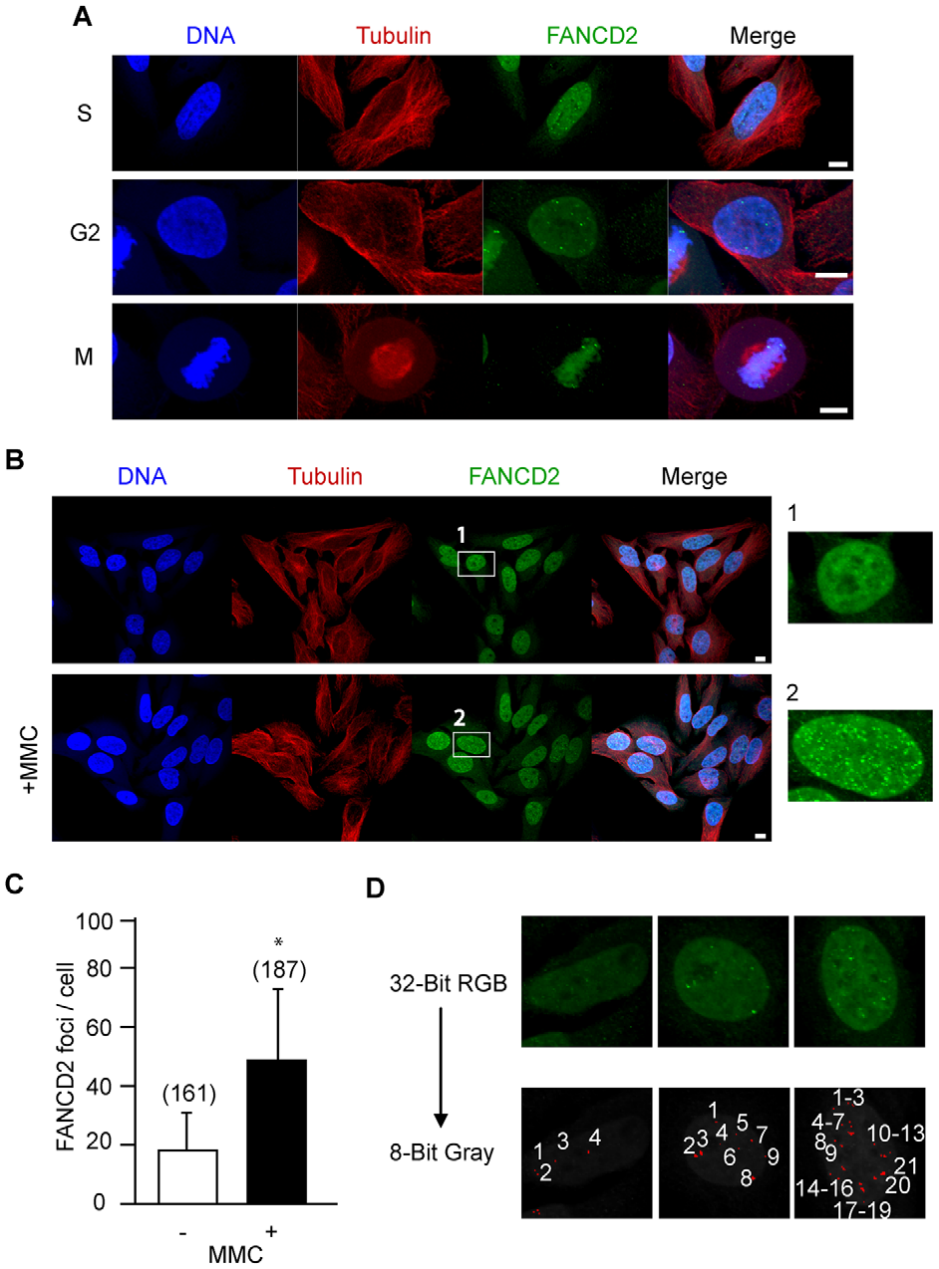
Interstrand crosslinking increases FANCD2 nuclear foci in HeLa cells. (A) FANCD2 immunostaining of synchronised HeLa cells in S-, G2- and M-phase. (B) FANCD2 nuclear foci during S-phase with or without MMC. (C) Nuclear FANCD2 foci counted per cell (mean ± standard deviation) from images in (B). In parentheses, total number of cells examined from 2 pooled replicates, *p<0.0001 (t-test). (D) Nuclear foci were calculated using a software-based approach. Confocal images were converted from 32-bit RGB to an 8-bit gray scale. ImageJ software was used to interrogate images, identify and count all nuclear foci of greater than 1.7x the mean nuclear background. In the 3 cells depicted the foci are numbered. (A, B) Scale bar, 10 μm.

ICLs, induced for example by MMC, are repaired by the FA pathway. It has been reported that in dividing somatic cells, FANCD2 localises to sites of such DNA damage during S-phase in the form of discrete nuclear foci [46], [47], [48], which remain on any sites of unrepaired damage during the subsequent cell cycle [49], [50]. Using synchronised HeLa cells, we confirmed this pattern of localisation (Fig 4A) and furthermore showed that FANCD2 foci increased nearly threefold following addition of 0.25 μg/ml MMC (Fig 4B–D). These observations are consistent with the ability of MMC to induce ICLs, and the involvement of the FA pathway in their repair.

**Figure 5:**
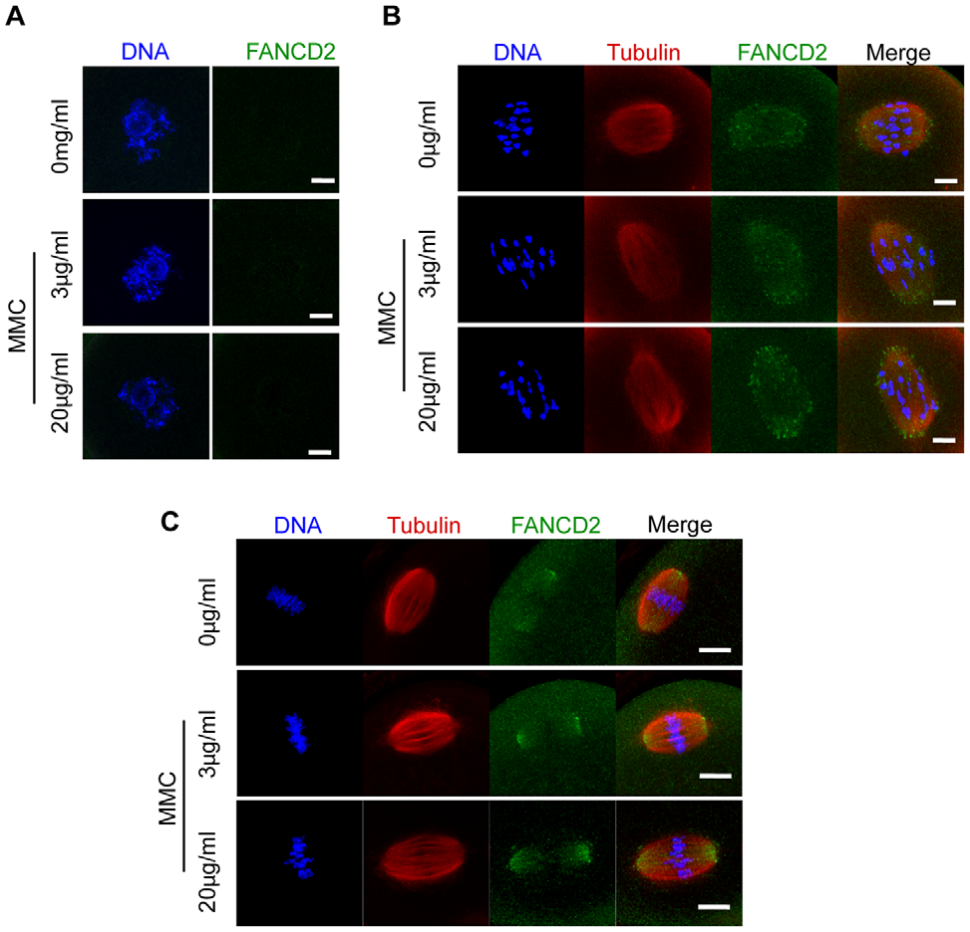
FANCD2 association with spindle microtubules and poles but not nuclear foci in oocytes. (A) FANCD2 immunostaining of the nuclei in GV oocytes with or without previous MMC addition at the concentrations stated. No FANCD2 foci were observed. (B, C) FANCD2 immunostaining of either meiotic spindles in meiosis I (B) or at metaphase II arrest (C). FANCD2 was present on spindle microtubules and poles, and its localisation was unaffected by MMC addition. Images are representative of at least 10 oocytes from at least two replicates. Scale bar, 10 μm.

In contrast to HeLa cells, GV oocytes showed little or no FANCD2 nuclear staining even following addition of 20 µg/ml MMC, which is 80 times the concentration used on HeLa cells (Fig 5A). This suggests that FANCD2 is not recruited to sites of DNA damage during GV arrest. Such a finding could simply be interpreted in terms of an absence of this FA pathway protein in oocytes, however FANCD2 immunostaining, with the same antibody used to label HeLa cells, was seen on both spindle microtubules and at spindle poles during both metaphase I and II (Fig 5B and 5C). Interestingly, its location was unaffected by prior treatment at the GV stage with MMC (Fig 5B and 5C).

**Figure 6:**
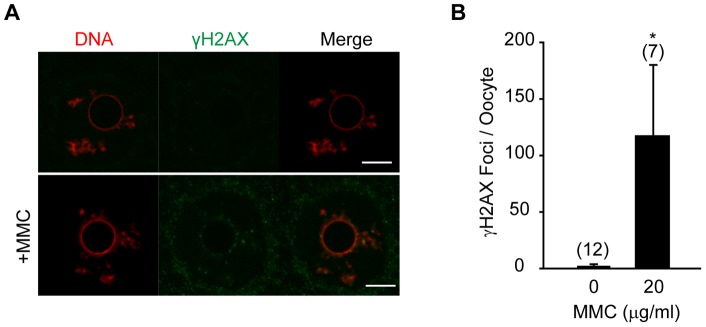
Induction of γH2AX foci in GV oocytes following MMC addition. (A) γH2AX immunostaining shown in GV oocytes treated with or without 20 µg/ml MMC. Scale bar, 10 μm. (B) γH2AX foci (mean ± standard deviation) counted per oocyte from images in (A). In parentheses, total number of oocytes examined, pooled from 2 independent experiments. *p<0.0001 (t-test).

To determine if GV oocytes can detect these ICLs, γH2AX immunostaining was performed. Without MMC treatment GV oocytes show little or no γH2AX foci, however, when GV oocytes were treated with MMC, discrete γH2AX foci were formed on chromatin (Fig 6A). Counting these foci revealed a 50-fold increase following addition of 20 μg/ml MMC (2 foci/oocyte vs 117 foci/oocyte; Fig 6B). Coupled with the FANCD2 localisation experiments, these observations indicate that oocytes can detect the ICLs lesions but the FA pathway is not recruited in response at the GV stage.

### FANCD2 foci in 1-cell embryos

**Figure 7:**
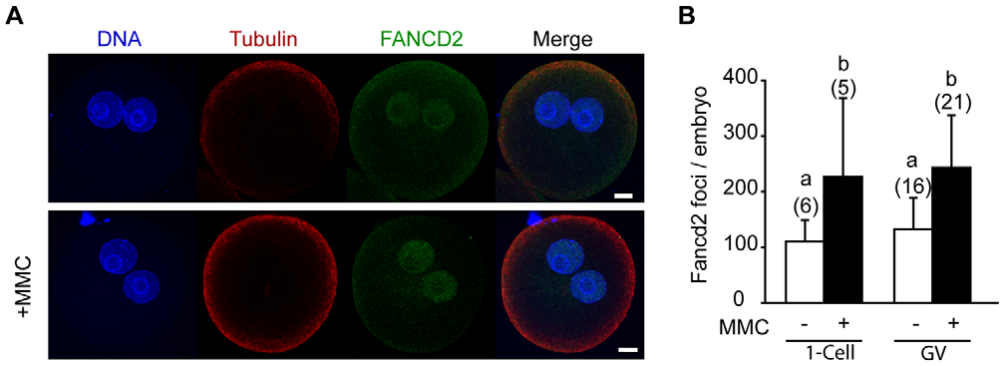
FANCD2 nuclear foci increase with ICLs in 1-cell embryos. (A) FANCD2 nuclear foci in 1-cell embryos treated with or without 3 µg/ml MMC. Scale bar, 10 μm. (B) Nuclear FANCD2 foci counted per embryo (mean ± standard deviation). MMC had been added at either GV stage oocytes, or to 1-cell embryos as indicated. Both groups had been in vitro matured and activated as described in Fig 3A. In parentheses, total number of embryos examined, pooled from 2 independent replicates. Different letters denote significantly difference (p<0.05; ANOVA, Tukey's post-hoc test).

As FANCD2 foci did not form during meiosis, we investigated if the FA pathway was only activated once embryogenesis was initiated. Parthenogenetically activated 1-cell embryos were challenged with or without MMC following pronucleus formation and the number of nuclear FANCD2 foci counted using the same protocol established for HeLa cells (Fig 4D). In the absence of MMC, FANCD2 was present as discrete foci on the pronuclei of the 1-cell embryo (Fig 7A). Further, the number of foci increased 2.5-fold, (110 nuclear foci/embryo vs 272 nuclear foci/embryo, p = 0.005; Fig 7B) following exposure to 3 μg/ml MMC. We note that this dose had no effect on foci formation when added to GV oocytes.

Finally, we examined if FANCD2 foci formed at a higher rate dependent on the time of MMC addition. Previously we had shown that MMC treatment at either the GV stage or 1-cell embryo stage both equally impaired embryo development (Fig 3). As such we anticipated that MMC addition at either the GV or 1-cell embryo stages would lead to the similar levels of DNA damage as defined by FANCD2 foci. Indeed, this proved to be the case with the same numbers of foci present with MMC independent of the time of addition (Fig 7B).

## Discussion

NCS, often used as an ionizing radiation mimetic to induce DSBs, was observed to be a potent inhibitor of meiotic maturation in mouse oocytes. Affected oocytes contained condensed and fragmented chromatin following just one hour exposure to the drug at the GV stage. The effects of the drug are presumed similar to IR, and indeed bovine oocytes demonstrate meiotic arrest following UV–C light exposure [19]. Based on these findings and those following treatment of whole ovary, we can surmise that DSBs profoundly affect GV oocytes at all follicular stages of their development. In primordial follicles it appears to promote loss through apoptosis, while in oocytes from fully-grown antral follicles, NCS-induced DSBs are an effective method of blocking meiotic maturation. The reason for the selective apoptosis in oocytes from primordial, but not antral follicles may be readily explained by the loss of TAp63, an oocyte-specific member of the p53 family essential for apoptosis, from oocytes following follicle recruitment [15], [16], [17].

Earlier studies have shown that IR-induced DSBs are detected in the newly formed 1-cell embryo [51], [52]. Mature sperm are thought to be incapable of identifying and repairing damaged DNA, and it is therefore left to the zygote formed immediately following gamete union to perform these tasks [53], [54]. The findings here, of MMC-induced FANCD2 foci in zygotes are consistent with the notion that DNA repair is active in early embryos, suggest that both ICLs and DSBs may be repaired at this point. Given the high rates of oxidative stress thought to be present in sperm [55], [56], the activation of the FA pathway at this time may of considerable benefit to the successful development of the embryo.

Women with FA have premature menopause [57], and consistent with this mouse knockouts for FA pathway members have a smaller ovarian reserve, which results in infertility later in life [58], [59], [60]. This represents the effect of an absent FA pathway during the mitotic S phases prior to meiosis, resulting in germ cell hypoplasia [61], [62], [63]. Indeed early meiotic functions for FA genes have recently been described also in zebrafish and C. elegans [64], [65]. However, the role of the FA pathway in oocytes, during later meiosis, has not been extensively investigated. Our data suggest that by the time oocytes have reached their fully-grown size and are arrested in the GV stage, then the FA pathway is not active. γH2AX foci however did form on treatment with MMC, consistent with previous observations of its recruitment to ICL sites. The recruitment of γH2AX but not FANCD2 suggests that although the FA pathway is not active, oocytes still have the capacity to recognise DNA damage. Such tagging of DNA at the GV stage may then be useful for its subsequent repair following egg activation. Instead, development of FANCD2 foci at sites of DNA damage does not occur until the 1-cell embryo stage. This increase in foci upon ICL induction indicates an active FA pathway in embryos. However, when embryos were treated with MMC, this led to decreased blastocyst rates even at low concentrations. This sensitivity to MMC in spite of increased FANCD2 foci should not imply an absence of ICL repair, given decreased cell survival is also observed in HeLa cells treated with similar MMC dosages [66], and these cells are well recognised to have a robust FA pathway [50], [67], [68]. Instead, the decreased survival of HeLa cells and the lowered blastocyst rates are both likely due to the severe DNA damage inflicted by MMC that overwhelms the repair process.

The lack of any effect in meiosis I and II upon MMC treatment, as well as the consequential defective blastocyst development were not necessarily unexpected. GV oocytes lack any gene transcription and do not replicate their DNA, which possibly explains the tolerance to high doses of MMC. In contrast, embryos need to undergo S-phases for proper growth, and it is likely that during these embryonic S-phases the FA pathway is full engaged. The GV stage, although being a time in which DNA damage is induced but not repaired, was not however uniquely vulnerable to ICL induced damage, given embryo development was equally impaired if 1-cell embryos were challenged with MMC at the same dose and for the same duration. Consistent with our observations are previous experiments on synchronised HeLa cells and fibroblasts where it was predominantly at S-phase that FANCD2 became recruited to DNA in an unperturbed cell cycle [45] and that in order for cells to become MMC sensitive they had to have undergone an S-phase [69]. However, severe MMC-induced DNA damage results in the recruitment of the ID complex, which FANCD2 is part of, to all nuclei irrespective of cell cycle phase [47]. More directly, others have demonstrated an ability of MMC to recruit the ID complex to sites of ICLs independent of replication [25], [26]. Furthermore, other studies point to activation of the FA pathway in response to DNA damage that might not be entirely the same as that induced in S-phase [70]. Therefore, the FA pathway is not exclusively S-phase based and replication dependent.

FANCD2 localisation to the meiosis I and II oocytes spindle and poles while initially surprising is similar to that reported previously for BRCA1 [71]. Indeed, these two proteins have been shown associated in somatic cells to sites of DNA damage [45], [46]. Given their non-chromosomal localisation in maturing oocytes, other cellular functions beyond DNA repair are possible. BRCA1 has been implicated in mitotic spindle integrity and function independent of repair [72], [73]. Consistent with a role for BRCA1 in spindle function, knockdown in mouse oocytes leads to spindle malformations and aneuploidy [71], [74]. It remains possible that FANCD2 has functions outside of DNA repair, and precedence for this is set by FANCD1 (BRCA2) which: (i) regulates the stability of BubR1, a member of the Spindle Assembly Checkpoint (SAC), that sets the timing of anaphase in mitosis [75] and (ii) regulates cytokinesis by localising to the central spindle formed during mitotic exit [68], [76], [77], [78]. Given the possibility of a non-repair function for FANCD2 suggested by its meiotic spindle association, future studies should therefore be directed, like for BRCA1, at its potential role in aneuploidy prevention.
